# 2,7-Dimeth­oxy-1,8-bis­(4-phen­oxy­benzo­yl)naphthalene

**DOI:** 10.1107/S1600536810042170

**Published:** 2010-10-23

**Authors:** Daichi Hijikata, Teruhisa Takada, Atsushi Nagasawa, Akiko Okamoto, Noriyuki Yonezawa

**Affiliations:** a Department of Organic and Polymer Materials Chemistry, Tokyo University of Agriculture & Technology, Koganei, Tokyo 184-8588, Japan

## Abstract

In the title mol­ecule {systematic name: [2,7-dimethoxy-8-(4-phenoxybenzoyl)naphthalen-1-yl](4-phenoxyphenyl)methan­one}, C_38_H_28_O_6_, the 4-phen­oxy­benzoyl units adopt a *syn* orientation with respect to the naphthalene ring system. The inter­nal benzene rings, *A* and *B*, make dihedral angles of 86.72 (5) and 79.22 (5)° with the naphthalene ring system. The two terminal benzene rings, *C* and *D*, of the 4-phen­oxy­benzoyl groups are twisted with respect to benzene rings *A* and *B*, with dihedral angles of *A*/*C* = 62.72 (8) and *B*/*D* = 87.61 (6)°. In the crystal, H atoms in the naphthalene system make two types of inter­molecular C—H⋯O inter­actions with the carbonyl O atom and the phenyl etheral O atom of neighbouring mol­ecules. Mol­ecules are further linked by C—H⋯π inter­actions involving a H atom of terminal benzene ring *D* and the π-system of the inter­nal benzene ring *A*, forming dimers centered about an inversion center.

## Related literature

For the syntheses of aroylated naphthalene compounds *via* electrophilic aromatic substitution of naphthalene derivatives, see: Okamoto & Yonezawa (2009 ▶). For the structures of closely related compounds, see: Nakaema *et al.* (2007 ▶, 2008 ▶); Mitsui *et al.* (2010 ▶); Muto *et al.* (2010 ▶); Watanabe *et al.* (2010**a* ▶,b* ▶).
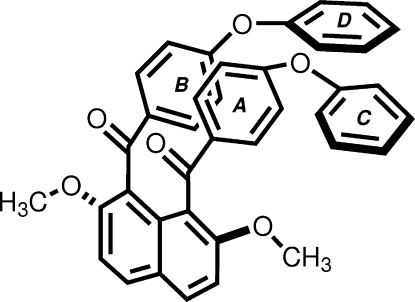


## Experimental

### 

#### Crystal data

C_38_H_28_O_6_
*M* *_r_* = 580.60
Monoclinic, 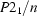

*a* = 12.0733 (4) Å
*b* = 12.4806 (4) Å
*c* = 19.8094 (6) Å
β = 91.115 (2)°
*V* = 2984.36 (15) Å^3^
*Z* = 4
Cu *K*α radiation
μ = 0.71 mm^−1^
*T* = 193 K
0.50 × 0.30 × 0.30 mm

#### Data collection

Rigaku R-AXIS RAPID diffractometer
Absorption correction: numerical (*NUMABS*; Higashi, 1999 ▶) *T*_min_ = 0.720, *T*_max_ = 0.816
54106 measured reflections
5458 independent reflections
4862 reflections with *I* > 2σ(*I*)
*R*_int_ = 0.038

#### Refinement

*R*[*F* ^2^ > 2σ(*F* ^2^)] = 0.036
*wR*(*F* ^2^) = 0.099
*S* = 1.04
5458 reflections
400 parameters
H-atom parameters constrained
Δρ_max_ = 0.16 e Å^−3^
Δρ_min_ = −0.13 e Å^−3^

### 

Data collection: *PROCESS-AUTO* (Rigaku, 1998 ▶); cell refinement: *PROCESS-AUTO*; data reduction: *CrystalStructure* (Rigaku/MSC, 2004 ▶); program(s) used to solve structure: *SIR2004* (Burla *et al.*, 2005 ▶); program(s) used to refine structure: *SHELXL97* (Sheldrick, 2008 ▶); molecular graphics: *ORTEPIII* (Burnett & Johnson, 1996 ▶); software used to prepare material for publication: *SHELXL97*.

## Supplementary Material

Crystal structure: contains datablocks I, global. DOI: 10.1107/S1600536810042170/su2217sup1.cif

Structure factors: contains datablocks I. DOI: 10.1107/S1600536810042170/su2217Isup2.hkl

Additional supplementary materials:  crystallographic information; 3D view; checkCIF report

## Figures and Tables

**Table 1:** Hydrogen-bond geometry (Å, °) *Cg*3 is the centroid of ring *A* (C12–C17).

*D*—H⋯*A*	*D*—H	H⋯*A*	*D*⋯*A*	*D*—H⋯*A*
C3—H3⋯O2^i^	0.95	2.44	3.1479 (17)	131
C6—H6⋯O6^ii^	0.95	2.56	3.3293 (16)	138
C35—H35⋯*Cg*3^iii^	0.95	2.78	3.6528 (17)	153

Symmetry codes: (i) 
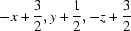
; (ii) 
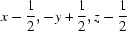
; (iii) 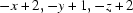.

**Fig. 1.**
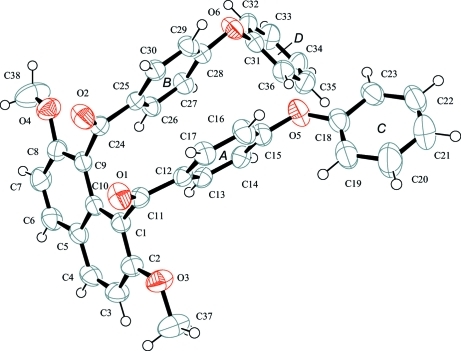
The molecular structure of the title molecule, showing 50% probability displacement ellipsoids.

**Fig. 2.**
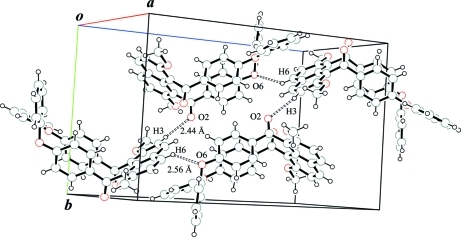
A partial crystal packing diagram of the title compound. The intermolecular C—H···O interactions are shown as double dashed lines (see Table 1 for details).

**Fig. 3.**
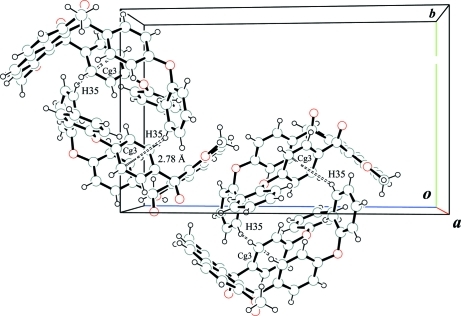
The dimeric pairs of the title molecule formed via C—H···π interactions, shown as double dashed lines (see Table 1 for details).

## supplementary crystallographic information

### Comment

In the course of our studies on selective electrophilic aromatic aroylation of 2,7-dimethoxynaphthalene, *peri*-aroylnaphthalene compounds have proved to be formed regioselectively by the aid of a suitable acidic mediator (Okamoto & Yonezawa, 2009). Recently, we reported on the crystal structures of three such compounds, 1,8-dibenzoyl-2,7-dimethoxynaphthalene (Nakaema *et al.*, 2008), bis(4-bromophenyl)(2,7-dimethoxynaphthalene-1,8-diyl)dimethanone (Watanabe *et al.*, 2010*a*), and 1,8-bis(4-methylbenzoyl)-2,7-dimethoxynaphthalene (Muto *et al.*, 2010). The aroyl groups at the 1,8-positions of the naphthalene rings in these compounds are twisted almost perpendicularly but a little tilted toward the *exo* sides against the naphthalene ring. In addition, 1,8-bis(4-chlorobenzoyl)-7-methoxynaphthalen-2-ol ethanol monosolvate (Mitsui *et al.*, 2010), which was formed by selective demethylation at the 2-methoxy group of 1,8-bis(4-chlorobenzoyl)-2,7-dimethoxynaphthalene (Nakaema *et al.*, 2007), solely has two *syn*-oriented chlorobenzoyl groups bonding to the naphthalene ring system, and in this molecule the 2-hydroxy group forms intramolecular hydrogen bonds with the carbonyl oxygen atom. As a part of our continuous studies on the molecular structures of this kind of homologous molecules, the crystal structure of the title compound, 1,8-bis(4-phenoxybenzoyl)-2,7-dimethoxynaphthalene, synthesized *via* nucleophilic aromatic substitution of (2,7-dimethoxynaphthalene-1,8-diyl)bis(4-fluorophenyl)dimethanone (Watanabe *et al.*, 2010*b*), is reported herein.

In the title molecule, illustrated in Fig. 1, two intervenient benzene rings, *A* (C12–C17) and *B* (C25–C30), are in a *syn* orientation with respect to the naphthalene ring system (C1–C10), and make dihedral angles of 86.72 (5) and 79.22 (5)°, respectively, with the naphthalene ring system. Furthermore, the dihedral angles between benzene rings *A* and *B* and the terminal benzene rings *C* (C18–C23) and *D* (C31–C36)] are *A*/*C* = 62.72 (8), *B*/*D* = 87.61 (6)°. Benzene rings *A* and *B* are configurated almost parallel to one another, the dihedral angle *A*/*B* being only 12.20 (6)°. On the other hand, benzene rings *C* and *D* are far from being parallel to one another with a dihedral angle *C*/*D* of 64.10 (8)°.

In the crystal, hydrogen atoms in the naphthalene ring form two types of intermolecular C—H···O interactions with the carbonyl oxygen atom (C3—H3···O2^i^ = 2.44 Å; see Fig. 2 and Table 1) and the phenyl ethereal oxygen atom (C6—H6···O6^ii^ = 2.56 Å; see Fig. 2 and Table 1). Moreover, molecules are linked by C—H···π interactions forming dimeric pairs. The terminal benzene ring *D* acts as a hydrogen-bond donor and the π system of the intervenient benzene ring *A* (with centroid Cg3) of an adjacent molecule acts as an acceptor (C35—H35···Cg3^iii^ = 2.78 Å; see Fig. 3 and Table 1).

### Experimental

In a 10 ml one-necked flask equipped with a condenser, (2,7-dimethoxynaphthalene-1,8-diyl)bis(4-fluorophenyl)dimethanone (1.0 mmol, 432.4 mg), phenol (4.0 mmol, 376.4 mg), potassium carbonate (8.0 mmol, 1.10 g) and freshly distilled DMAc (2.0 ml) were stirred at 423 K for 6 h. This mixture was then added dropwise into methanol (20 ml) resulting in the formation of a pale yellow precipitate. The crude material was purified by column chromatography (silica gel, hexane: AcOEt= 2:1) to give the title compound (yield 104 mg, 18%). The isolated product was recrystallized from acetone to give block-like yellow single-crystals of the title compound. M.p. 441.6–444.4 K; ^1^HNMR δ (300 MHz, CDCl_3_): 3.72 (6*H*, s), 6.82–6.94 (4*H*, m), 7.09 (4*H*, d, J=7.5 Hz), 7.14–7.21 (4*H*, m), 7.36 (4*H*, t, J=8.4 Hz), 7.55–7.78 (4*H*,m), 7.92 (2*H*, d, J=7.5 Hz) p.p.m.; ^13^CNMR δ (75 MHz, CDCl_3_): 56.442, 111.128, 116.575, 120.274, 121.440, 124.345, 125.415, 129.467, 129.850, 131.360, 131.876, 133.424, 155.405, 155.998, 161.474, 195.057 p.p.m.; IR (KBr): 1673 (C=O), 1267 (Ar—O—Me) cm^-1^; HRMS (m/*z*): [*M* + H]^+^ calcd for C_38_H_29_O_6_, 581.1964 found, 581.82.

### Refinement

All H atoms were found in a difference Fourier map and were subsequently refined as riding atoms: C—H = 0.95 (aromatic) and 0.98 (methyl) Å, with U_iso_(H) = 1.2 *U*_eq_(C).

### Crystal data

**Table:**

C_38_H_28_O_6_	*F*(000) = 1216
*M**_r_* = 580.60	*D*_x_ = 1.292 Mg m^−^^3^
Monoclinic, *P*2_1_/*n*	Cu *K*α radiation, λ = 1.54187 Å
Hall symbol: -P 2yn	Cell parameters from 37382 reflections
*a* = 12.0733 (4) Å	θ = 3.5–68.3°
*b* = 12.4806 (4) Å	µ = 0.71 mm^−^^1^
*c* = 19.8094 (6) Å	*T* = 193 K
β = 91.115 (2)°	Block, yellow
*V* = 2984.36 (15) Å^3^	0.50 × 0.30 × 0.30 mm
*Z* = 4	

### Data collection

**Table:**

Rigaku R-AXIS RAPID diffractometer	5458 independent reflections
Radiation source: rotating anode	4862 reflections with *I* > 2σ(*I*)
graphite	*R*_int_ = 0.038
Detector resolution: 10.00 pixels mm^-1^	θ_max_ = 68.3°, θ_min_ = 4.2°
ω scans	*h* = −14→14
Absorption correction: numerical (*NUMABS*; Higashi, 1999)	*k* = −15→15
*T*_min_ = 0.720, *T*_max_ = 0.816	*l* = −23→23
54106 measured reflections	

### Refinement

**Table:**

Refinement on *F*^2^	Secondary atom site location: difference Fourier map
Least-squares matrix: full	Hydrogen site location: inferred from neighbouring sites
*R*[*F*^2^ > 2σ(*F*^2^)] = 0.036	H-atom parameters constrained
*wR*(*F*^2^) = 0.099	*w* = 1/[σ^2^(*F*_o_^2^) + (0.0496*P*)^2^ + 0.5589*P*] where *P* = (*F*_o_^2^ + 2*F*_c_^2^)/3
*S* = 1.03	(Δ/σ)_max_ = 0.001
5458 reflections	Δρ_max_ = 0.16 e Å^−^^3^
400 parameters	Δρ_min_ = −0.13 e Å^−^^3^
0 restraints	Extinction correction: *SHELXL97* (Sheldrick, 2008), Fc^*^=kFc[1+0.001xFc^2^λ^3^/sin(2θ)]^-1/4^
Primary atom site location: structure-invariant direct methods	Extinction coefficient: 0.00337 (19)

### Special details

**Table:**

Geometry. All e.s.d.'s (except the e.s.d. in the dihedral angle between two l.s. planes) are estimated using the full covariance matrix. The cell e.s.d.'s are taken into account individually in the estimation of e.s.d.'s in distances, angles and torsion angles; correlations between e.s.d.'s in cell parameters are only used when they are defined by crystal symmetry. An approximate (isotropic) treatment of cell e.s.d.'s is used for estimating e.s.d.'s involving l.s. planes.
Refinement. Refinement of *F*^2^ against ALL reflections. The weighted *R*-factor *wR* and goodness of fit *S* are based on *F*^2^, conventional *R*-factors *R* are based on *F*, with *F* set to zero for negative *F*^2^. The threshold expression of *F*^2^ > σ(*F*^2^) is used only for calculating *R*-factors(gt) *etc*. and is not relevant to the choice of reflections for refinement. *R*-factors based on *F*^2^ are statistically about twice as large as those based on *F*, and *R*- factors based on ALL data will be even larger.

### Fractional atomic coordinates and isotropic or equivalent isotropic displacement parameters (Å^2^)

**Table:**

	*x*	*y*	*z*	*U*_iso_*/*U*_eq_	
O1	0.96053 (8)	0.06684 (7)	0.81736 (5)	0.0546 (2)	
O2	0.77701 (9)	−0.00287 (7)	0.90988 (5)	0.0586 (3)	
O3	0.97983 (8)	0.27793 (8)	0.73618 (5)	0.0584 (3)	
O4	0.51924 (8)	0.06935 (9)	0.91941 (5)	0.0615 (3)	
O5	1.08799 (8)	0.36651 (9)	1.06682 (5)	0.0620 (3)	
O6	0.79851 (8)	0.28865 (8)	1.16825 (4)	0.0556 (3)	
C1	0.83140 (10)	0.20657 (9)	0.79447 (6)	0.0406 (3)	
C2	0.86712 (11)	0.27048 (10)	0.74219 (6)	0.0471 (3)	
C3	0.79253 (12)	0.32401 (11)	0.69846 (7)	0.0522 (3)	
H3	0.8189	0.3692	0.6637	0.063*	
C4	0.68212 (12)	0.30989 (11)	0.70687 (6)	0.0510 (3)	
H4	0.6314	0.3447	0.6769	0.061*	
C5	0.64066 (10)	0.24509 (10)	0.75887 (6)	0.0447 (3)	
C6	0.52610 (11)	0.22967 (12)	0.76518 (7)	0.0542 (3)	
H6	0.4772	0.2613	0.7328	0.065*	
C7	0.48293 (11)	0.17099 (13)	0.81612 (7)	0.0579 (4)	
H7	0.4053	0.1600	0.8186	0.069*	
C8	0.55556 (11)	0.12678 (11)	0.86504 (7)	0.0493 (3)	
C9	0.66870 (10)	0.13650 (9)	0.86044 (6)	0.0408 (3)	
C10	0.71611 (10)	0.19418 (9)	0.80557 (6)	0.0394 (3)	
C11	0.92353 (10)	0.15289 (9)	0.83493 (6)	0.0405 (3)	
C12	0.96973 (9)	0.21139 (9)	0.89451 (6)	0.0388 (3)	
C13	0.92476 (9)	0.30783 (9)	0.91570 (6)	0.0407 (3)	
H13	0.8647	0.3388	0.8909	0.049*	
C14	0.96670 (10)	0.35918 (10)	0.97258 (6)	0.0446 (3)	
H14	0.9354	0.4250	0.9870	0.054*	
C15	1.05418 (10)	0.31414 (11)	1.00819 (6)	0.0478 (3)	
C16	1.10038 (11)	0.21766 (11)	0.98832 (7)	0.0535 (3)	
H16	1.1600	0.1867	1.0135	0.064*	
C17	1.05836 (11)	0.16749 (10)	0.93132 (7)	0.0477 (3)	
H17	1.0903	0.1020	0.9169	0.057*	
C18	1.19700 (12)	0.39961 (11)	1.07515 (7)	0.0522 (3)	
C19	1.27352 (14)	0.39668 (15)	1.02490 (8)	0.0715 (4)	
H19	1.2535	0.3709	0.9812	0.086*	
C20	1.38027 (17)	0.4318 (2)	1.03878 (11)	0.0967 (7)	
H20	1.4343	0.4283	1.0046	0.116*	
C21	1.40910 (18)	0.47185 (18)	1.10142 (11)	0.0946 (6)	
H21	1.4825	0.4962	1.1105	0.113*	
C22	1.33065 (17)	0.47633 (14)	1.15093 (9)	0.0758 (5)	
H22	1.3501	0.5042	1.1942	0.091*	
C23	1.22396 (14)	0.44059 (11)	1.13820 (7)	0.0602 (4)	
H23	1.1699	0.4441	1.1723	0.072*	
C24	0.73586 (10)	0.08501 (9)	0.91703 (6)	0.0412 (3)	
C25	0.74864 (9)	0.14370 (9)	0.98181 (6)	0.0388 (3)	
C26	0.69332 (10)	0.23842 (10)	0.99588 (6)	0.0423 (3)	
H26	0.6442	0.2685	0.9629	0.051*	
C27	0.70890 (10)	0.28958 (11)	1.05731 (6)	0.0458 (3)	
H27	0.6706	0.3542	1.0667	0.055*	
C28	0.78094 (10)	0.24554 (10)	1.10496 (6)	0.0441 (3)	
C29	0.83819 (11)	0.15178 (10)	1.09148 (6)	0.0487 (3)	
H29	0.8880	0.1224	1.1242	0.058*	
C30	0.82200 (11)	0.10197 (10)	1.03023 (6)	0.0461 (3)	
H30	0.8614	0.0380	1.0207	0.055*	
C31	0.77444 (11)	0.39731 (11)	1.17747 (6)	0.0477 (3)	
C32	0.68244 (12)	0.42435 (12)	1.21339 (7)	0.0569 (4)	
H32	0.6344	0.3705	1.2300	0.068*	
C33	0.66113 (13)	0.53178 (13)	1.22497 (8)	0.0651 (4)	
H33	0.5974	0.5521	1.2493	0.078*	
C34	0.73172 (13)	0.60918 (13)	1.20139 (8)	0.0637 (4)	
H34	0.7169	0.6827	1.2097	0.076*	
C35	0.82377 (13)	0.58022 (13)	1.16573 (8)	0.0625 (4)	
H35	0.8722	0.6339	1.1494	0.075*	
C36	0.84602 (12)	0.47327 (12)	1.15348 (7)	0.0561 (3)	
H36	0.9095	0.4528	1.1290	0.067*	
C37	1.02198 (14)	0.34475 (15)	0.68454 (9)	0.0741 (5)	
H37A	1.1031	0.3439	0.6867	0.089*	
H37B	0.9955	0.4182	0.6909	0.089*	
H37C	0.9964	0.3182	0.6404	0.089*	
C38	0.40630 (15)	0.0786 (2)	0.93694 (12)	0.1008 (7)	
H38A	0.3948	0.0437	0.9806	0.121*	
H38B	0.3598	0.0439	0.9023	0.121*	
H38C	0.3863	0.1545	0.9401	0.121*	

### Atomic displacement parameters (Å^2^)

**Table:**

	*U*^11^	*U*^22^	*U*^33^	*U*^12^	*U*^13^	*U*^23^
O1	0.0598 (6)	0.0431 (5)	0.0604 (6)	0.0113 (4)	−0.0108 (4)	−0.0065 (4)
O2	0.0817 (7)	0.0420 (5)	0.0514 (5)	0.0130 (5)	−0.0147 (5)	−0.0058 (4)
O3	0.0499 (5)	0.0617 (6)	0.0636 (6)	0.0012 (4)	0.0039 (4)	0.0206 (5)
O4	0.0488 (5)	0.0698 (7)	0.0658 (6)	−0.0101 (5)	0.0012 (4)	0.0149 (5)
O5	0.0549 (6)	0.0761 (7)	0.0546 (6)	−0.0006 (5)	−0.0087 (4)	−0.0167 (5)
O6	0.0719 (6)	0.0533 (6)	0.0411 (5)	0.0076 (5)	−0.0123 (4)	−0.0064 (4)
C1	0.0459 (6)	0.0357 (6)	0.0399 (6)	0.0017 (5)	−0.0052 (5)	0.0007 (5)
C2	0.0492 (7)	0.0438 (7)	0.0481 (7)	0.0032 (5)	−0.0007 (5)	0.0045 (5)
C3	0.0610 (8)	0.0491 (8)	0.0462 (7)	0.0042 (6)	−0.0033 (6)	0.0116 (6)
C4	0.0593 (8)	0.0491 (7)	0.0440 (7)	0.0091 (6)	−0.0120 (6)	0.0050 (6)
C5	0.0491 (7)	0.0430 (7)	0.0414 (6)	0.0049 (5)	−0.0106 (5)	−0.0018 (5)
C6	0.0494 (7)	0.0581 (8)	0.0545 (8)	0.0052 (6)	−0.0163 (6)	0.0021 (6)
C7	0.0407 (7)	0.0666 (9)	0.0660 (9)	−0.0031 (6)	−0.0106 (6)	0.0031 (7)
C8	0.0481 (7)	0.0477 (7)	0.0519 (7)	−0.0055 (6)	−0.0038 (6)	0.0015 (6)
C9	0.0445 (6)	0.0374 (6)	0.0400 (6)	−0.0013 (5)	−0.0073 (5)	−0.0025 (5)
C10	0.0452 (6)	0.0351 (6)	0.0377 (6)	0.0013 (5)	−0.0081 (5)	−0.0038 (5)
C11	0.0411 (6)	0.0356 (6)	0.0448 (6)	0.0009 (5)	−0.0015 (5)	0.0042 (5)
C12	0.0381 (6)	0.0349 (6)	0.0433 (6)	−0.0012 (5)	−0.0016 (5)	0.0054 (5)
C13	0.0369 (6)	0.0359 (6)	0.0492 (7)	−0.0001 (5)	−0.0035 (5)	0.0058 (5)
C14	0.0423 (6)	0.0381 (6)	0.0535 (7)	−0.0013 (5)	0.0008 (5)	−0.0017 (5)
C15	0.0455 (7)	0.0513 (7)	0.0465 (7)	−0.0035 (6)	−0.0041 (5)	−0.0029 (6)
C16	0.0509 (7)	0.0552 (8)	0.0537 (8)	0.0085 (6)	−0.0150 (6)	0.0020 (6)
C17	0.0497 (7)	0.0411 (7)	0.0521 (7)	0.0088 (5)	−0.0073 (6)	0.0027 (5)
C18	0.0560 (8)	0.0464 (7)	0.0534 (7)	0.0011 (6)	−0.0158 (6)	0.0014 (6)
C19	0.0657 (10)	0.0865 (12)	0.0619 (9)	−0.0152 (8)	−0.0117 (7)	−0.0112 (8)
C20	0.0701 (11)	0.1271 (18)	0.0927 (14)	−0.0304 (12)	−0.0038 (10)	−0.0231 (13)
C21	0.0769 (12)	0.1078 (16)	0.0979 (15)	−0.0283 (11)	−0.0281 (11)	−0.0112 (12)
C22	0.0966 (13)	0.0629 (10)	0.0665 (10)	−0.0146 (9)	−0.0363 (10)	−0.0002 (8)
C23	0.0814 (10)	0.0475 (8)	0.0508 (8)	−0.0021 (7)	−0.0179 (7)	0.0023 (6)
C24	0.0459 (6)	0.0358 (6)	0.0418 (6)	−0.0033 (5)	−0.0029 (5)	0.0021 (5)
C25	0.0422 (6)	0.0348 (6)	0.0392 (6)	−0.0049 (5)	−0.0026 (5)	0.0039 (5)
C26	0.0403 (6)	0.0453 (7)	0.0411 (6)	0.0026 (5)	−0.0060 (5)	0.0010 (5)
C27	0.0447 (6)	0.0462 (7)	0.0464 (7)	0.0065 (5)	−0.0040 (5)	−0.0048 (5)
C28	0.0478 (7)	0.0461 (7)	0.0382 (6)	−0.0042 (5)	−0.0034 (5)	−0.0021 (5)
C29	0.0580 (8)	0.0434 (7)	0.0441 (7)	0.0031 (6)	−0.0138 (6)	0.0043 (5)
C30	0.0567 (7)	0.0348 (6)	0.0463 (7)	0.0020 (5)	−0.0076 (5)	0.0034 (5)
C31	0.0519 (7)	0.0522 (8)	0.0387 (6)	−0.0001 (6)	−0.0077 (5)	−0.0075 (5)
C32	0.0554 (8)	0.0596 (9)	0.0558 (8)	−0.0123 (7)	0.0045 (6)	−0.0102 (7)
C33	0.0582 (8)	0.0671 (10)	0.0706 (10)	−0.0053 (7)	0.0151 (7)	−0.0195 (8)
C34	0.0663 (9)	0.0533 (8)	0.0719 (10)	−0.0056 (7)	0.0077 (7)	−0.0161 (7)
C35	0.0629 (9)	0.0602 (9)	0.0646 (9)	−0.0141 (7)	0.0077 (7)	−0.0052 (7)
C36	0.0517 (8)	0.0661 (9)	0.0506 (7)	−0.0023 (7)	0.0063 (6)	−0.0075 (7)
C37	0.0649 (9)	0.0804 (11)	0.0774 (11)	0.0013 (8)	0.0147 (8)	0.0301 (9)
C38	0.0584 (10)	0.1314 (19)	0.1134 (16)	−0.0008 (11)	0.0215 (10)	0.0495 (14)

### Geometric parameters (Å, °)

**Table:**

O1—C11	1.2167 (14)	C18—C23	1.3826 (19)
O2—C24	1.2135 (15)	C19—C20	1.384 (2)
O3—C2	1.3714 (16)	C19—H19	0.9500
O3—C37	1.4217 (17)	C20—C21	1.376 (3)
O4—C8	1.3727 (16)	C20—H20	0.9500
O4—C38	1.418 (2)	C21—C22	1.378 (3)
O5—C18	1.3863 (17)	C21—H21	0.9500
O5—C15	1.3871 (15)	C22—C23	1.382 (2)
O6—C28	1.3771 (14)	C22—H22	0.9500
O6—C31	1.3996 (16)	C23—H23	0.9500
C1—C2	1.3829 (17)	C24—C25	1.4832 (16)
C1—C10	1.4219 (17)	C25—C26	1.3887 (17)
C1—C11	1.5145 (16)	C25—C30	1.3938 (16)
C2—C3	1.4061 (18)	C26—C27	1.3839 (17)
C3—C4	1.358 (2)	C26—H26	0.9500
C3—H3	0.9500	C27—C28	1.3847 (17)
C4—C5	1.4092 (19)	C27—H27	0.9500
C4—H4	0.9500	C28—C29	1.3877 (18)
C5—C6	1.4045 (19)	C29—C30	1.3738 (18)
C5—C10	1.4339 (16)	C29—H29	0.9500
C6—C7	1.359 (2)	C30—H30	0.9500
C6—H6	0.9500	C31—C32	1.3729 (19)
C7—C8	1.4070 (19)	C31—C36	1.374 (2)
C7—H7	0.9500	C32—C33	1.385 (2)
C8—C9	1.3761 (17)	C32—H32	0.9500
C9—C10	1.4323 (17)	C33—C34	1.376 (2)
C9—C24	1.5139 (16)	C33—H33	0.9500
C11—C12	1.4871 (16)	C34—C35	1.377 (2)
C12—C13	1.3887 (17)	C34—H34	0.9500
C12—C17	1.3952 (16)	C35—C36	1.384 (2)
C13—C14	1.3838 (17)	C35—H35	0.9500
C13—H13	0.9500	C36—H36	0.9500
C14—C15	1.3783 (18)	C37—H37A	0.9800
C14—H14	0.9500	C37—H37B	0.9800
C15—C16	1.3873 (19)	C37—H37C	0.9800
C16—C17	1.3792 (18)	C38—H38A	0.9800
C16—H16	0.9500	C38—H38B	0.9800
C17—H17	0.9500	C38—H38C	0.9800
C18—C19	1.372 (2)		
			
C2—O3—C37	118.15 (11)	C21—C20—H20	119.6
C8—O4—C38	118.24 (12)	C19—C20—H20	119.6
C18—O5—C15	120.22 (11)	C20—C21—C22	119.42 (18)
C28—O6—C31	117.95 (10)	C20—C21—H21	120.3
C2—C1—C10	119.93 (11)	C22—C21—H21	120.3
C2—C1—C11	114.52 (11)	C21—C22—C23	120.55 (16)
C10—C1—C11	125.54 (10)	C21—C22—H22	119.7
O3—C2—C1	115.37 (11)	C23—C22—H22	119.7
O3—C2—C3	122.61 (12)	C22—C23—C18	119.11 (17)
C1—C2—C3	122.01 (12)	C22—C23—H23	120.4
C4—C3—C2	118.77 (12)	C18—C23—H23	120.4
C4—C3—H3	120.6	O2—C24—C25	120.76 (11)
C2—C3—H3	120.6	O2—C24—C9	120.79 (11)
C3—C4—C5	121.84 (12)	C25—C24—C9	118.44 (10)
C3—C4—H4	119.1	C26—C25—C30	118.80 (11)
C5—C4—H4	119.1	C26—C25—C24	123.49 (10)
C6—C5—C4	120.53 (11)	C30—C25—C24	117.70 (11)
C6—C5—C10	119.75 (12)	C27—C26—C25	120.77 (11)
C4—C5—C10	119.71 (12)	C27—C26—H26	119.6
C7—C6—C5	122.17 (12)	C25—C26—H26	119.6
C7—C6—H6	118.9	C26—C27—C28	119.28 (12)
C5—C6—H6	118.9	C26—C27—H27	120.4
C6—C7—C8	118.68 (13)	C28—C27—H27	120.4
C6—C7—H7	120.7	O6—C28—C27	123.30 (11)
C8—C7—H7	120.7	O6—C28—C29	115.86 (11)
O4—C8—C9	115.52 (11)	C27—C28—C29	120.81 (11)
O4—C8—C7	122.74 (12)	C30—C29—C28	119.26 (11)
C9—C8—C7	121.73 (12)	C30—C29—H29	120.4
C8—C9—C10	120.43 (11)	C28—C29—H29	120.4
C8—C9—C24	115.57 (11)	C29—C30—C25	121.08 (12)
C10—C9—C24	124.00 (10)	C29—C30—H30	119.5
C1—C10—C9	125.36 (10)	C25—C30—H30	119.5
C1—C10—C5	117.62 (11)	C32—C31—C36	122.03 (13)
C9—C10—C5	117.00 (11)	C32—C31—O6	118.55 (13)
O1—C11—C12	121.75 (11)	C36—C31—O6	119.34 (12)
O1—C11—C1	120.60 (11)	C31—C32—C33	118.59 (14)
C12—C11—C1	117.59 (10)	C31—C32—H32	120.7
C13—C12—C17	118.87 (11)	C33—C32—H32	120.7
C13—C12—C11	121.50 (10)	C34—C33—C32	120.34 (14)
C17—C12—C11	119.60 (11)	C34—C33—H33	119.8
C14—C13—C12	120.57 (11)	C32—C33—H33	119.8
C14—C13—H13	119.7	C35—C34—C33	120.08 (15)
C12—C13—H13	119.7	C35—C34—H34	120.0
C15—C14—C13	119.50 (12)	C33—C34—H34	120.0
C15—C14—H14	120.2	C34—C35—C36	120.31 (14)
C13—C14—H14	120.2	C34—C35—H35	119.8
C14—C15—O5	116.51 (12)	C36—C35—H35	119.8
C14—C15—C16	121.14 (12)	C31—C36—C35	118.64 (13)
O5—C15—C16	122.21 (12)	C31—C36—H36	120.7
C17—C16—C15	118.87 (12)	C35—C36—H36	120.7
C17—C16—H16	120.6	O3—C37—H37A	109.5
C15—C16—H16	120.6	O3—C37—H37B	109.5
C16—C17—C12	121.04 (12)	H37A—C37—H37B	109.5
C16—C17—H17	119.5	O3—C37—H37C	109.5
C12—C17—H17	119.5	H37A—C37—H37C	109.5
C19—C18—C23	121.03 (14)	H37B—C37—H37C	109.5
C19—C18—O5	123.84 (12)	O4—C38—H38A	109.5
C23—C18—O5	115.09 (13)	O4—C38—H38B	109.5
C18—C19—C20	119.02 (16)	H38A—C38—H38B	109.5
C18—C19—H19	120.5	O4—C38—H38C	109.5
C20—C19—H19	120.5	H38A—C38—H38C	109.5
C21—C20—C19	120.8 (2)	H38B—C38—H38C	109.5
			
C37—O3—C2—C1	−178.33 (13)	C18—O5—C15—C14	−123.87 (13)
C37—O3—C2—C3	1.6 (2)	C18—O5—C15—C16	60.33 (18)
C10—C1—C2—O3	179.42 (11)	C14—C15—C16—C17	0.9 (2)
C11—C1—C2—O3	−1.79 (16)	O5—C15—C16—C17	176.54 (12)
C10—C1—C2—C3	−0.50 (19)	C15—C16—C17—C12	−1.0 (2)
C11—C1—C2—C3	178.30 (12)	C13—C12—C17—C16	0.78 (19)
O3—C2—C3—C4	178.22 (12)	C11—C12—C17—C16	−177.51 (12)
C1—C2—C3—C4	−1.9 (2)	C15—O5—C18—C19	8.8 (2)
C2—C3—C4—C5	1.3 (2)	C15—O5—C18—C23	−173.45 (12)
C3—C4—C5—C6	−178.26 (13)	C23—C18—C19—C20	2.4 (3)
C3—C4—C5—C10	1.5 (2)	O5—C18—C19—C20	179.97 (17)
C4—C5—C6—C7	−177.53 (14)	C18—C19—C20—C21	−1.7 (3)
C10—C5—C6—C7	2.7 (2)	C19—C20—C21—C22	0.3 (4)
C5—C6—C7—C8	1.7 (2)	C20—C21—C22—C23	0.3 (3)
C38—O4—C8—C9	164.34 (16)	C21—C22—C23—C18	0.4 (3)
C38—O4—C8—C7	−17.1 (2)	C19—C18—C23—C22	−1.7 (2)
C6—C7—C8—O4	177.86 (13)	O5—C18—C23—C22	−179.52 (13)
C6—C7—C8—C9	−3.7 (2)	C8—C9—C24—O2	98.63 (15)
O4—C8—C9—C10	179.63 (11)	C10—C9—C24—O2	−82.66 (16)
C7—C8—C9—C10	1.0 (2)	C8—C9—C24—C25	−80.37 (14)
O4—C8—C9—C24	−1.60 (17)	C10—C9—C24—C25	98.34 (14)
C7—C8—C9—C24	179.81 (12)	O2—C24—C25—C26	−172.30 (12)
C2—C1—C10—C9	−175.50 (12)	C9—C24—C25—C26	6.70 (17)
C11—C1—C10—C9	5.84 (19)	O2—C24—C25—C30	9.04 (18)
C2—C1—C10—C5	3.26 (17)	C9—C24—C25—C30	−171.96 (11)
C11—C1—C10—C5	−175.39 (11)	C30—C25—C26—C27	−1.23 (18)
C8—C9—C10—C1	−177.92 (12)	C24—C25—C26—C27	−179.88 (11)
C24—C9—C10—C1	3.42 (19)	C25—C26—C27—C28	0.25 (19)
C8—C9—C10—C5	3.31 (17)	C31—O6—C28—C27	−22.47 (18)
C24—C9—C10—C5	−175.35 (11)	C31—O6—C28—C29	159.22 (12)
C6—C5—C10—C1	176.01 (11)	C26—C27—C28—O6	−177.55 (11)
C4—C5—C10—C1	−3.76 (17)	C26—C27—C28—C29	0.68 (19)
C6—C5—C10—C9	−5.12 (17)	O6—C28—C29—C30	177.76 (12)
C4—C5—C10—C9	175.10 (11)	C27—C28—C29—C30	−0.6 (2)
C2—C1—C11—O1	−87.22 (15)	C28—C29—C30—C25	−0.4 (2)
C10—C1—C11—O1	91.50 (16)	C26—C25—C30—C29	1.33 (19)
C2—C1—C11—C12	90.10 (13)	C24—C25—C30—C29	−179.95 (12)
C10—C1—C11—C12	−91.18 (14)	C28—O6—C31—C32	107.42 (14)
O1—C11—C12—C13	−177.79 (11)	C28—O6—C31—C36	−75.86 (15)
C1—C11—C12—C13	4.92 (16)	C36—C31—C32—C33	0.7 (2)
O1—C11—C12—C17	0.44 (18)	O6—C31—C32—C33	177.33 (13)
C1—C11—C12—C17	−176.85 (11)	C31—C32—C33—C34	−0.7 (2)
C17—C12—C13—C14	−0.39 (18)	C32—C33—C34—C35	0.4 (3)
C11—C12—C13—C14	177.86 (11)	C33—C34—C35—C36	−0.1 (2)
C12—C13—C14—C15	0.28 (18)	C32—C31—C36—C35	−0.4 (2)
C13—C14—C15—O5	−176.41 (11)	O6—C31—C36—C35	−177.04 (12)
C13—C14—C15—C16	−0.6 (2)	C34—C35—C36—C31	0.1 (2)

### Hydrogen-bond geometry (Å, °)

**Table:**

Cg3 is the centroid of ring *A* (C12–C17).

**Table:**

*D*—H···*A*	*D*—H	H···*A*	*D*···*A*	*D*—H···*A*
C3—H3···O2^i^	0.95	2.44	3.1479 (17)	131
C6—H6···O6^ii^	0.95	2.56	3.3293 (16)	138
C35—H35···Cg3^iii^	0.95	2.78	3.6528 (17)	153

Symmetry codes: (i) −*x*+3/2, *y*+1/2, −*z*+3/2; (ii) *x*−1/2, −*y*+1/2, *z*−1/2; (iii) −*x*+2, −*y*+1, −*z*+2.
